# Benzalkonium Chloride-Coated Iron Oxide Nanoparticles: Cytotoxic Potential on Murine Leydig Cells (TM3)

**DOI:** 10.1021/acsomega.5c09618

**Published:** 2025-12-10

**Authors:** Pedro Igor Macário Viana, Thalita Marcolan Valverde, Daniele Alves Fagundes, Carla Cristina Martins Silva, Graziela de Paula Ferreira Dantas, Larissa Kennedy Moreira Freitas, Danilo Roberto Carvalho Ferreira, Bruna de Paula Dias, Pedro Lana Gastelois, Clascídia Aparecida Furtado, José Domingos Ardisson, Ângela Leão Andrade, Guilherme Mattos Jardim Costa

**Affiliations:** 1 Laboratório de Biologia Celular, Departamento de Morfologia, Instituto de Ciências Biológicas (ICB-UFMG), 28114 Universidade Federal de Minas Gerais, Belo Horizonte, MG 31270-901, Brazil; 2 Departamento de Química, Instituto de Ciências Exatas e Biológicas (ICEB/UFOP), 28115 Universidade Federal de Ouro Preto, Ouro Preto, MG 35400-000, Brazil; 3 Laboratório de Física Aplicada, 54530 Centro de Desenvolvimento da Tecnologia Nuclear (CDTN/CNEN), Belo Horizonte, MG 31270-901, Brazil; 4 Laboratório de Química de Nanoestruturas de Carbono, 54530 Centro de Desenvolvimento da Tecnologia Nuclear (CDTN/CNEN), Belo Horizonte, MG 31270-901, Brazil; 5 Centro de Desenvolvimento da Tecnologia Nuclear (CDTN/CNEN), Belo Horizonte, MG 31270-901, Brazil

## Abstract

This study presents a novel nanocomplex, Mag-Bc, combining magnetite (Mag) and benzalkonium chloride (Bc). The objective was to evaluate the cytotoxic potential of Mag-Bc across three cell lines (TM3, VERO, and AML-12) and to assess its antiandrogenic effects specifically on TM3 cells. Physicochemical characterization confirmed the formation of partially oxidized magnetite and successful Bc coating, which conferred a positive charge to the nanoparticles. Viability assays on TM3 cells demonstrated no reduction below 70% at any dose or exposure time. Cellular internalization studies revealed efficient uptake of both coated and uncoated nanoparticles, indicating that the Bc coating did not impair cellular internalization. The nanocomplex exhibited intermediate superoxide anion production, maintaining levels comparable to control groups and demonstrating superior biocompatibility compared to magnetite and benzalkonium chloride alone. Testosterone levels remained unchanged following exposure to the Bc-coated nanoparticles. Collectively, these findings demonstrate that the Mag-Bc nanocomplex exhibits enhanced biocompatibility, characterized by a maintained cell viability, preserved cellular internalization capacity, reduced oxidative stress, and unchanged endocrine function. In contrast, cell growth kinetics of TM3 cells exposed to Bc-coated nanoparticles demonstrated reduced proliferation between days 3 and 8 compared with controls, suggesting potential time-dependent effects. The growth inhibition of Leydig cells induced by the nanocomplex may have implications for applications requiring suppression of testicular cell proliferation or for the development of nanoparticle-based therapeutic strategies targeting cell growth arrest.

## Graphical abstract

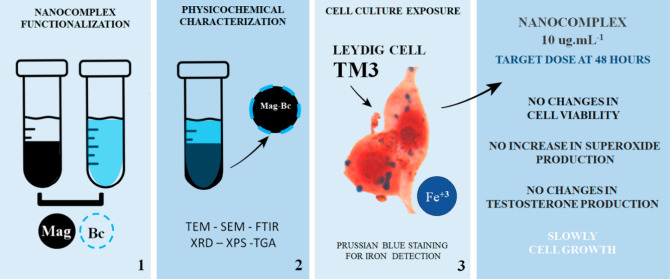

## 1 Introduction

Male fertility depends on a balance of molecular and cellular processes in which micronutrients play essential roles. Among them, iron plays a crucial role in supporting male fertility.^1^ Spermatogenesis is an iron-dependent process, acting in different mechanisms, including DNA synthesis and cellular metabolism.^2^ However, its imbalance can disrupt testicular homeostasis and trigger cell death, leading to ferroptosis, an iron-dependent form of nonapoptotic cell death.^3,4^ Exposure to large amounts of iron, particularly when associated with iron-containing nanomaterials, can increase degeneration of spermatogenic cells, desquamation of germ cells, and vacuolization of seminiferous tubules.^5,6^

Nanobiotechnology is advancing the applications of different nanomaterials in biomedicine.^7^ Nanoparticles, among the nanomaterials, offer flexibility in their applications, displaying diverse physicochemical characteristics that can be explored in a range of biological systems. Iron oxide nanoparticles (IONPs) can be synthesized in different ways, and their physicochemical properties can be modulated according to specific conditions.^8^ This is important for the development of new therapy approaches, as some physicochemical characteristics are directly linked to biocompatibility.^8^ While these features make IONPs promising candidates for personalized therapies, their interaction with the reproductive system remains poorly understood.

Our research group recently investigated the biocompatibility and biodistribution of IONPs in the testicular environment.^9,10^ In general, it was observed that Leydig cells have a high affinity for IONPs, compromising their steroidogenic activity and, consequently, testosterone production. Leydig cells are responsible for the production of testosterone, which will act on androgen receptors in Sertoli cells on the seminiferous epithelium.^11^ Therefore, any dissociation of it can negatively affect the testosterone produced by acting on germ cell development during spermatogenesis.^12^

Nanoparticle coating/functionalization has several applications, like improving their stability and targeting, where coatings can carry bioactive molecules or ligands.^6,13^ In this context, IONPs can deliver and enhance the effect of chemical compounds to generate an adverse effect on spermatogenesis. Benzalkonium chloride (Bc) rises as an alternative to a nanoparticle functionalization agent. BC is a quaternary ammonium compound, Food and Drug Administration-approved (FDA), used in personal care products, household, and industrial cleaning products.^14^ However, Bc is being investigated for its potential to impact human and animal fertility,^15–17^ particularly through its decellularization potential.^18^ While the mechanisms remain unclear, quaternary ammonium compounds and iron oxide nanoparticles (IONPs) are both linked to hormonal synthesis disruption, and studying their combined effects could reveal potential interactions affecting Leydig cells.

The mechanism underlying nanoparticle-induced male reproductive dysfunction is gaining attention as a promising area of research.^9,19^ However, the safety of these nanomaterials in the testicular environment should be the primary focus of new approaches to nanoparticle-based therapies. In this context, the primary objective of this study was to assess the cytotoxic potential of benzalkonium chloride-coated iron oxide nanoparticles (Mag-Bc) on Leydig cells *in vitro* and evaluate their antiandrogenic effects to determine their potential for therapeutic application in the male reproductive tract. The cytotoxicity of Mag-Bc was assessed on TM3 cultures, regarding the: (i) synthesis, functionalization, and characterization of the Mag-Bc nanocomplex; (ii) cytotoxic profile on TM3 Leydig cell cultures and comparison with other mammalian immortalized cell lines; (iii) cellular internalization of Mag-Bc nanoparticles; (iv) oxidative stress induction through superoxide production and its impact on testosterone secretion; and (v) effects on cell growth kinetics. These investigations aim to clarify the potential risks and therapeutic implications of Mag-Bc on Leydig cells.

## 2 Results and Discussion

### 2.1 Physicochemical Characterization

The nanocrystalline structure of the synthesized IONPs was identified by Rietveld refinement of the X-ray diffraction (XRD) patterns. The pseudo–Voigt function was used, and the resulting Rietveld profiles are displayed in Figure 1a–a’’. This identification involving the Mag (a), Mag-Bc005 (a’), and Mag-Bc01 (a’’) samples was performed with space group *Fd*3̅*m*. The quality of the Rietveld refinement was evaluated from the profile factor (*R*_p_), weighted profile factor (*R*_wp_), goodness of fit indicator (*S*), Bragg Factor (*R*_B_), and crystallographic *R*_F_-factor (*R*_F_) parameters (Table 1). The high *R*_wp_ values (39–47%) are expected for nanostructured samples due to peak broadening and the amorphous background. However, the *R*_wp_/*R* exp ratios (0.88–0.99) confirm the refinements are statistically reliable.

**Table 1:** Structural Parameters and Rietveld Agreement Factors for the Mag, Mag-Bc005, and Mag-Bc01 Samples

	samples		
parameter	Mag	Mag-Bc005	Mag-Bc01
magnetite	8.356 ± 0.001	8.357 ± 0.001	8.355 ± 0.001
	8.356 ± 0.001	8.357 ± 0.001	8.355 ± 0.001
	8.356 ± 0.001	8.357 ± 0.001	8.355 ± 0.001
*R*_P_	0.314	0.368	0.361
*R*_wp_	0.396	0.467	0.464
*S*	0.88	0.93	0.99
*R*_B_	0.139	1.72	1.84
*R*_F_	0.151	2.07	2.05

**Figure 1:**
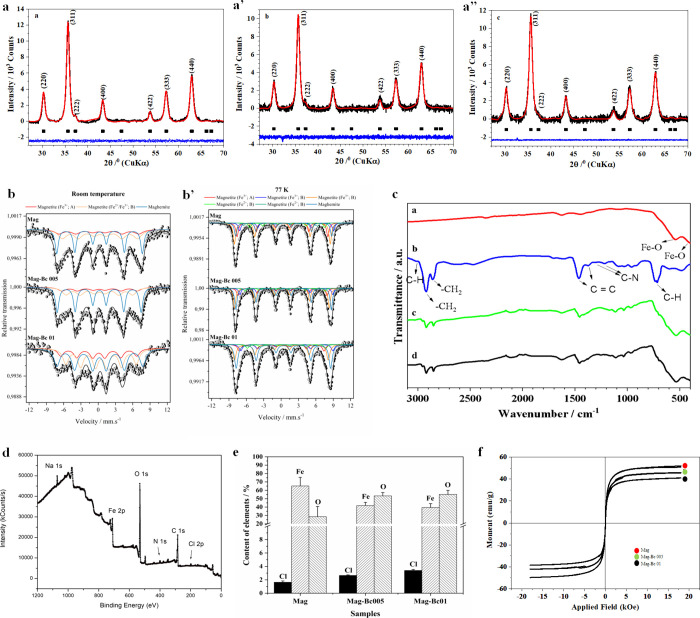
Characterization of the nanoparticles. (a–a’’) Powder X-ray diffraction patterns (black) of the samples: Mag (a), Mag-Bc005 (a’), and Mag-Bc01 (a’’). The solid (red) lines correspond to the profiles fitted through Rietveld refinement. (b, b’) Mössbauer analysis of Mag, Mag-Bc005, and Mag-Bc01 at room temperature (b) and 77 K (b’). (c) FTIR spectra of the samples Mag (a—red), Bc (b—blue), Mag-Bc005 (c—green), and Mag-Bc01 (d—black). (d) XPS spectrum for the Mag-Bc005 sample to confirm Bc coating. (e) X-ray fluorescence element content in the samples: Mag, Mag-Bc005, and Mag-Bc01. (f) Magnetization hysteresis curves measured at 300 K for the samples: Mag (red), Mag-Bc005 (green), and Mag-Bc01 (black).

The unit cell parameter *a*_0_ ≈ 8.39 Å is typical of stoichiometric bulk magnetite, while the cubic maghemite has *a*_0_ ≈ 8.34 Å.^20^ On the other hand, partially oxidized magnetite nanoparticles may exhibit smaller lattice parameters of 8.38 Å ≥ *a*_0_ ≥ 8.34 Å.^21^ In this work, we observed the occurrence of a single crystallographic phase of partially oxidized magnetite for all samples (Table 1).

Fe Mössbauer spectra obtained at room temperature (RT) and 77 K are shown in Figure 1b,b’. At RT (b), the spectra could be fitted with two sextets corresponding to magnetite and an additional sextet attributed to maghemite, confirming the partial oxidation of the nanoparticles. The spectra exhibit broadened lines, which can be ascribed to the collapse of hyperfine fields at the tetrahedral (A) and octahedral (B) sites of magnetite structure, a feature typically associated with superparamagnetic relaxation of IONPs.^22^ When the temperature was lowered to 77 K (b’), the relaxation effects observed were mostly suppressed, allowing the observation of more detailed information about the sample’s hyperfine characteristics. At this temperature, below the Verwey transition, the two magnetite sextets evolved into five, consistent with the charge ordering characteristic of magnetite.^23^ The hyperfine parameters (Supplementary Tables 1–6) are in agreement with values reported in the literature, reinforcing the phase identification obtained. The presence of maghemite was estimated with relative fractions of 21% for Mag. It is noteworthy that even at 77 K, some nanoparticles still exhibit superparamagnetic relaxation, as evidenced by residual line broadening.

**Figure 2:**
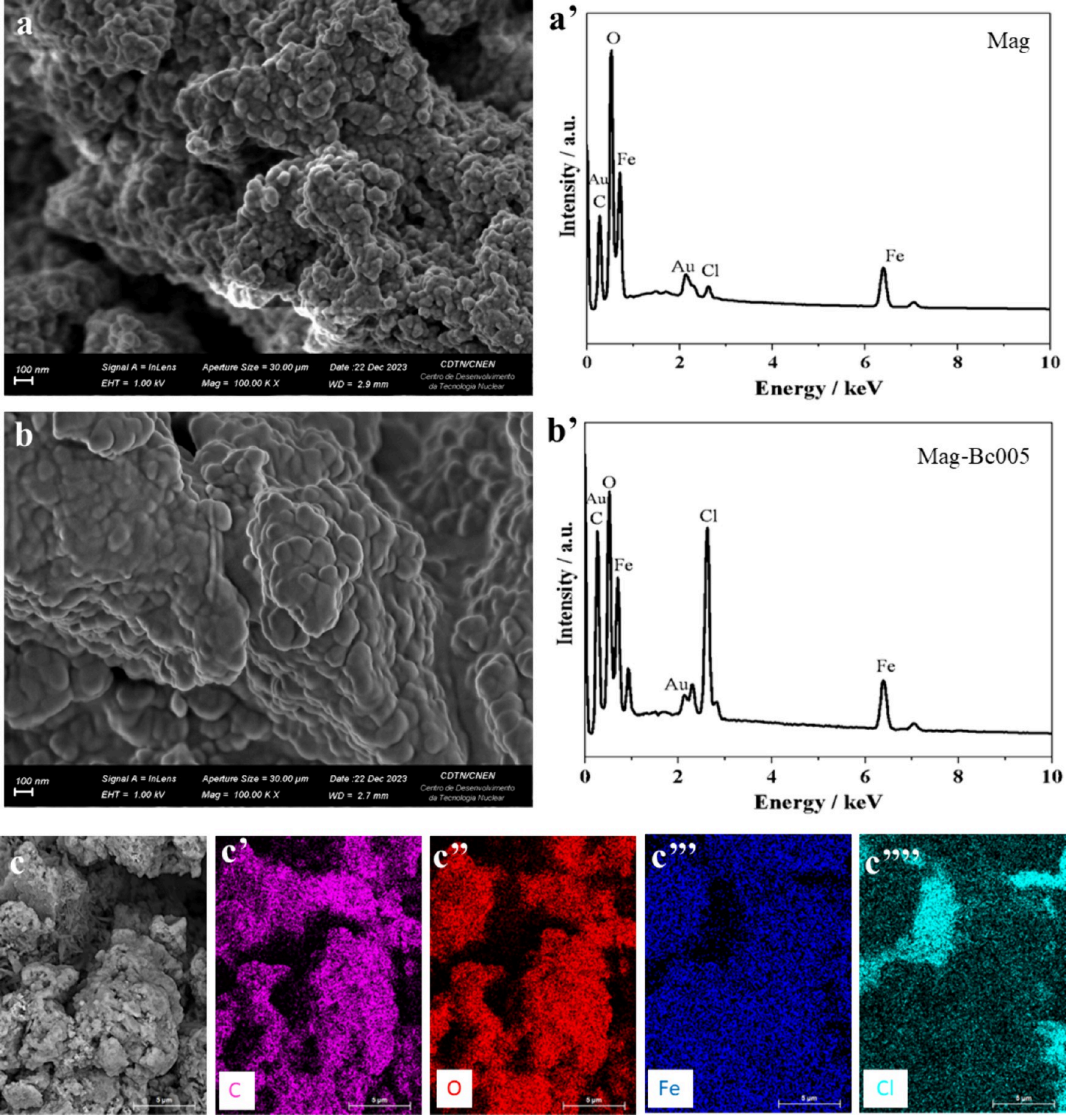
Scanning electron microscopy and energy-dispersive spectroscopy analysis of Mag and Mag-Bc005 samples. SEM micrographs (a, b) and the EDS spectra (a’, b’) for the samples Mag (a, a’) and Mag-Bc005 (b, b’). (c–c’’’’) Elemental mapping to visualize the spatial distribution of elements for the sample Mag-Bc005, showing in sequence carbon (c’), oxygen (c’’), iron (c’’’), and chlorine (c’’’’).

The Fourier Transform Infrared (FTIR) spectra of the Mag, Bc, Mag-Bc005, and Mag-Bc01 samples are shown in Figure 1c. The FTIR data (Figure 1c) support the predominant presence of a magnetite phase in the Mag sample (red line). According to the literature,^24,25^ the FTIR spectrum of magnetite exhibits two strong infrared absorption bands. The band position of ν_1_ is in the range of 540–590 cm^–1^, while that of ν_2_ is in the range of 428–467 cm^–1^. The band position of v1 is assigned to the stretching vibration Fe–O bonds among the tetrahedral A-sites, while v2 is assigned to Fe–O bonds among the octahedral B-sites.^26^ In the spectrum of Bc (blue line), the C–H stretching vibrations of symmetric and asymmetric −CH_2_ on the carbon chain are represented by the peaks at 2920 and 2850 cm^–1^, respectively. The absorption peaks between 1483 and 1456 cm^–1^ are due to the skeleton stretching vibration of C=C in the benzene ring. The stretching vibration of C–N causes peaks at 1374–1218 cm^–1^. The stretching and out-of-plane bending vibration of C–H in the benzene ring evoke peaks at 3019 and 880–658 cm^–1^, respectively. The attached water contributes to −OH’s stretching and bending vibrations at 3430 and 1635 cm^–1^.^27–29^

The FTIR spectra (Figure 1c) of the Mag-Bc005 (green line) and Mag-Bc01 (black line) samples also show the band characteristic of the magnetite as well as the transmittance bands typical of benzalkonium chloride, proving that the peptizing agent has been strongly adsorbed at the surface of the nanoparticles. However, the band at 1236 cm^–1^ (vibration of C–N) of benzalkonium chloride was shifted to 1274 cm^–1^. The shift in this band indicates an intermolecular interaction. The X-ray photoelectron spectroscopy (XPS) spectrum obtained for the Mag-Bc005 sample (Figure 1d) confirms the presence of Fe 2p, O 1s, N 1s, and Cl 2p signals, evidencing the successful coating of benzalkonium chloride on the magnetite surface. The high surface sensitivity of XPS allows the clear identification of these elements, supporting the functionalization of partially oxidized magnetite and demonstrating the stability and efficacy of the benzalkonium chloride layer in the nanocomplex.

Elemental analysis was performed using an X-ray fluorescence analysis (Figure 1e). It was found that Cl content increased, and the Fe content decreased with the increase in the benzalkonium chloride concentration in the sample. The decrease in Fe signal could indicate that the benzalkonium chloride coated the magnetite nanoparticles in the sample. The surface charge of the nanoparticles was determined by zeta potential (ZP) measurements. The Mag sample showed a negative zeta potential of −8.3 mV. Following coating the Mag-Bc005 and Mag-Bc01 samples with Bc, the ZP increased to 33.6 and 35.7 mV as a result of the interaction of the magnetite in aqueous dispersion with Bc. Dynamic light scattering (DLS) revealed that the Mag-Bc005 formulation was 323.2 nm in solution, and the polydispersity index was 0.274, considered moderately monodisperse. The current formulation is optimized for *in vitro* mechanistic studies; however, the optimization of suspension stability would be necessary for future systemic delivery and *in vivo* applications.

This result indicates that magnetite has been effectively coated by benzalkonium chloride. Such a positive ZP is due to the presence of a positively charged amino group of benzalkonium on the surface of the Mag-Bc005 and Mag-Bc01 samples. The relatively high surface potential of the coated samples could play a critical role in minimizing the aggregation of particles and improving the colloidal stability of the suspension. Particles of the same charge type with high absolute ZP tend to repel each other, hindering their aggregation. Conventionally, high ZP values fit outside the range between −30 and +30 mV.^30^

Magnetization curves are presented in Figure 1f, obtained for Mag (red), Mag-Bc005 (green), and Mag-Bc01­(black) samples at 300 K. There was no hysteresis in the magnetization for all samples, suggesting that the magnetic particles produced are superparamagnetic. The saturation magnetization (*M*_S_) was obtained from moment versus magnetic field (B) curves. For the Mag sample, the *M*_S_ value is 52.1 emu g^–1^, being higher than values reported in the literature (46.3 emu g^–1^) for samples prepared by the same method^31^ and lower than the bulk value, 90 emu g^–1^.^32^ For the samples coated with Bc, Mag-Bc 005, and Mag-Bc 01, the corresponding *M*_S_ values are 46.0 and 41.0 emu g^–1^. The decrease in the *M*_S_ value for the samples coated can be attributed to the presence of a nonmagnetic layer on the surface of magnetite nanoparticles or the direct attachment of amine groups to the iron ions that could result in the pinning of the surface magnetic spins.^33^

### 2.2 Electron Microscopy on Nanocomplex Characterization

Scanning electron microscopy (SEM) images (Figures 2a and 2b) show the samples Mag and Mag-Bc005, respectively. Comparing the elemental analysis obtained by energy-dispersive X-ray spectroscopy (EDS) of Mag (Figure 2a’) and Mag-Bc005 (Figure 2b’), we can observe that the peaks of C and Cl increased, while the Fe peak decreased. This indicates that the surface of the mag sample was coated with Bc, as seen in the elemental map for Mag-Bc005 (Figure 2c–c’’’’). The nitrogen (N) peak was not detected by EDS due to the low sensitivity of the method to light elements and the ultrathin organic nature of the benzalkonium chloride coating, which resulted in a nitrogen signal below the detection limit. In contrast, the presence of Cl Kα (2.62 keV) serves as a reliable marker of BC functionalization.

Figure 3 shows the high-resolution TEM (HRTEM) images of Mag (a) and Mag-Bc005 (b) and their respective particle size distributions (Figure 3c,d). The coated magnetite sample and the uncoated one showed equivalent particle size, with average diameters for the Mag 8.7 ± 2.2 nm and Mag-Bc005 8.5 ± 2.1 nm, showing no statistically significant differences. The selected area electron diffraction (SAED) data from both samples confirm the magnetite phase. SAED patterns (Figure 3e,f) were further analyzed using JEMS software (v. 3.4922U2010). The experimental SAED patterns were compared with simulated electron diffraction profiles for standard magnetite (*a* = 8.3967 Å) and maghemite (*a* = 8.33 Å), both with a centric setting space group, *Fd3̅*m*. For such simulations, an acceleration voltage of 200 kV, a size 6 nm crystal, and a Lorentzian model for line shape were chosen. The results are in agreement with the standard magnetite sample used as a reference.

**Figure 3:**
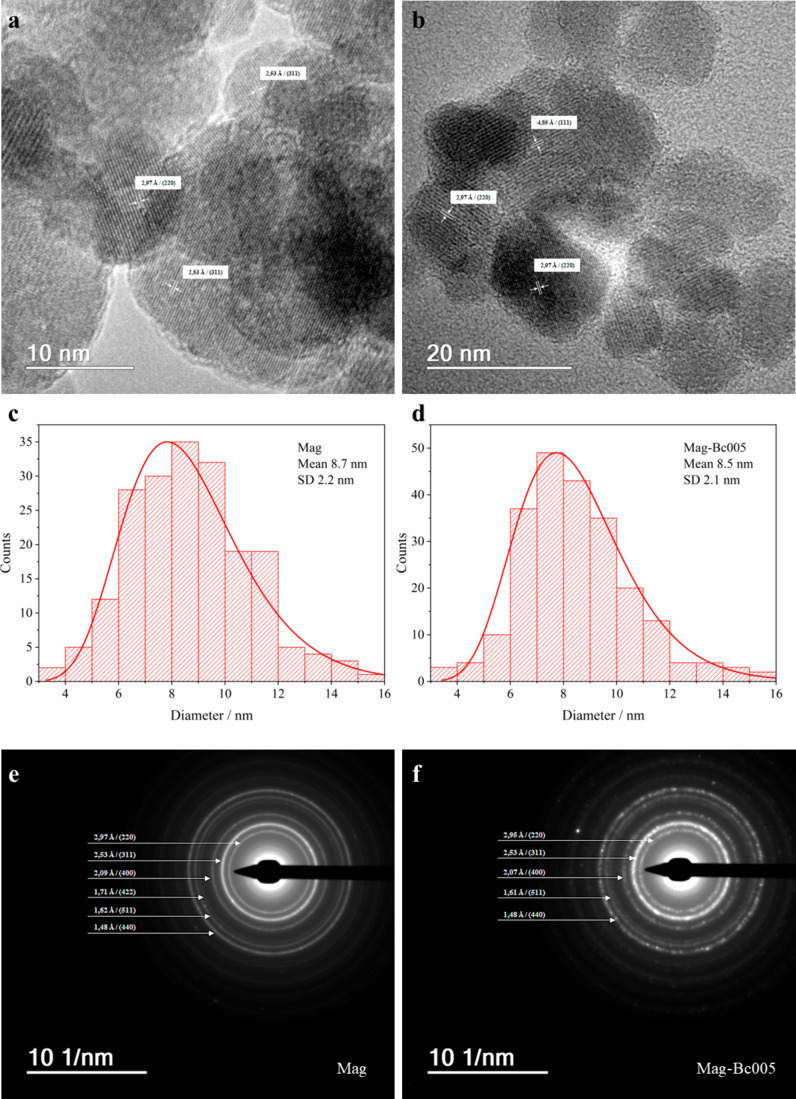
High-resolution transmission electron microscopy characterization of Mag and Mag-Bc005 samples. (a, b) HRTEM images showing crystallographic planes (white arrows) for the Mag (a) and Mag-Bc005 (b). (c, d) Histograms corresponding to particle size and distributions for Mag (c) and Mag-Bc005 (d). (e, f) SAED patterns for Mag (e) and Mag-Bc005 (f) samples.

### 2.3 Thermogravimetric Analysis (TGA)

The TGA profiles are shown in Figure 4 for Bc (a), Mag (b), and Mag-Bc005 (c). From these data, the iron oxide content was determined. The TGA curve for the Bc (Figure 4a) exhibits two mass loss steps, the first of ∼5.0% between ∼50 and 105 °C and a second one of ∼94.0% from 105 up to 300 °C. This resulted in an almost 100% weight loss, possibly attributed to the loss of moisture and pyrolysis of the benzalkonium chloride during heating, which were consistent with other studies.^27,28^

**Figure 4:**
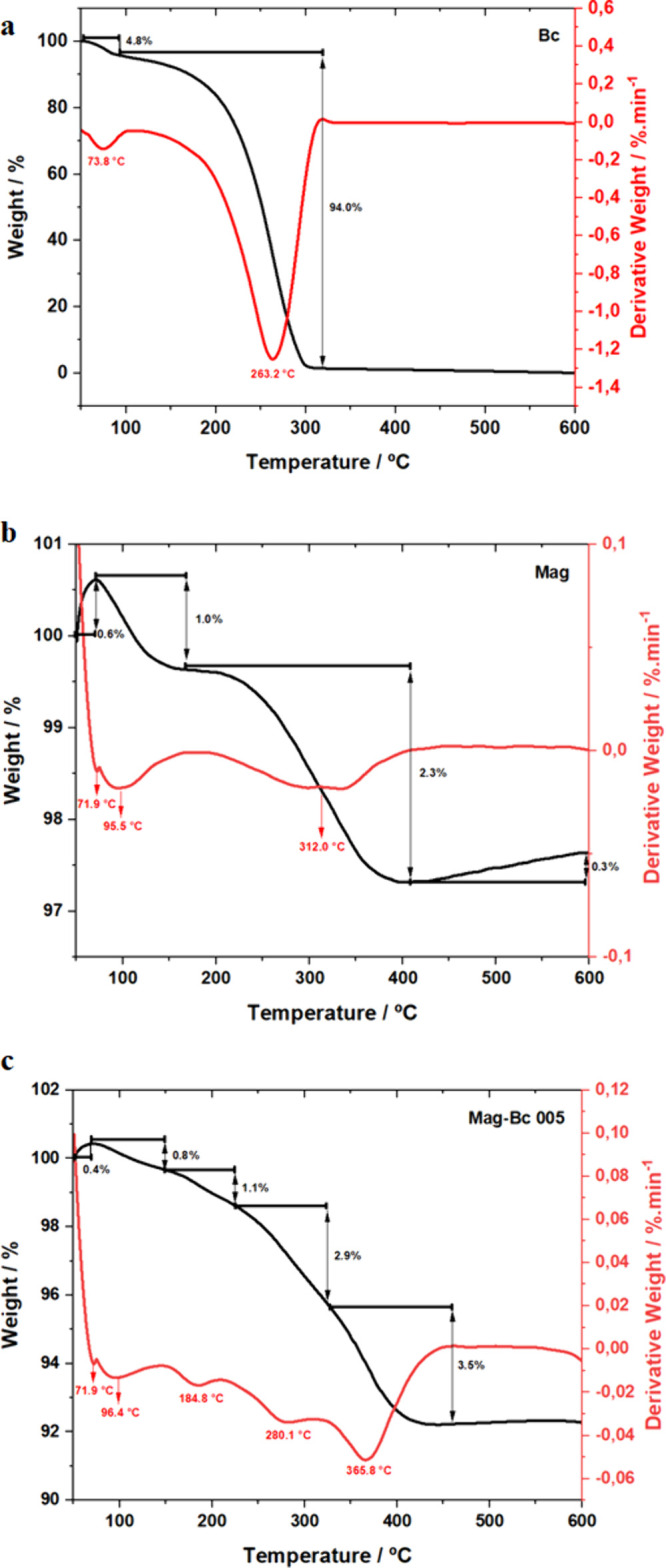
Thermogravimetric and derivative thermogravimetry curves for Bc, Mag, and Mag-Bc005. TGA (black lines) and DTG (red lines) curves for the decomposition of the samples Bc (a), Mag (b), and Mag-Bc005 (c).

The TGA curve of the Mag sample shows a well-defined thermal event with a mass loss of approximately 1.0% in the temperature interval of 70–160 °C (Figure 4b). This thermal event is attributed to the loss of the physically adsorbed water molecules. In the temperature range of 160–400 °C, magnetite is oxidized to maghemite, simultaneously with the desorption of strongly bound hydroxyl groups from the nanoparticle surfaces, resulting in an approximate mass loss of 2.3%.^34,35^ The next thermal event observed between 400 and 590 °C is attributed to the oxidation of maghemite into hematite, which is reflected by a small mass gain of approximately 0.3%.^34,35^

In the TGA plot of the Mag-Bc005 (Figure 4c), the first mass loss (0.8%), which occurs in the range of 70–145 °C, is related to the loss of water adsorbed on the surface of the samples. The second (1.1%) stage, between 145 and 225 °C, can be interpreted as being due to more strong bound water molecules. In the sequence, the sample exhibited a 2.9% mass loss event around 280 °C. Considering that the Mag sample also exhibits a loss of ∼2.3% in a similar temperature range, the ∼0.6% difference can be attributed to the decomposition of Bc fractions adsorbed on the nanoparticle surface. The event initiated around 360 °C (with a loss of ∼3.5%) may be related to the decomposition of more stable Bc, possibly due to the pyrolysis of alkyl or aromatic groups.^36^

The small initial mass gain observed at temperatures below 100 °C for the Mag (Figure 4b) and Mag-Bc005 (Figure 4c) samples is consistent with surface oxidation or chemisorption of O_2_ on magnetite nanoparticles.^37,38^ More insights about the thermal decomposition peaks can be obtained from DTG curves, as shown in Figure 4 in red. The Bc decomposition peak at about 260 °C was shifted to ∼280–366 °C in the Mag-Bc005 sample, indicating a higher thermal stability of the Bc-loaded sample in comparison to Bc. This increase in the thermal stability of Mag-Bc005 suggests a chemical interaction between the components of the drug with the magnetite, confirming the FTIR result.^39^

### 2.4 Turbidity and Precipitate Formation as a Test of UV Efficiency

After incubation, to verify UV radiation efficiency, the medium was evaluated for turbidity and precipitation at 24 and 48 h (Supplementary Figure 1). Only the positive control (Luria–Bertani (LB) medium + *E. coli*) showed turbidity and precipitation on the bottom as a result of bacterial growth. The other tubes with magnetite (Mag) and the nanocomplex (Mag-Bc005) did not show any changes in turbidity and precipitation, confirming the UV radiation efficiency (Table 2).

**Table 2:** Positive (+) and Negative (−) Results for Turbidity and Precipitate Formation in the Medium with Mag, Mag-Bc005, after UV Radiation Exposure, and Negative and Positive Controls

samples	24 h	48 h
Mag	–	–
Mag-Bc005	–	–
control	–	–
positive control (*E. coli*)	+	+

### 2.5 Cell Viability Assay with VERO, AML-12, and TM3 Cell Lines

The VERO cell line, derived from kidney tissue, when exposed to the uncoated nanoparticle, Mag, showed no reduction in viability at 24 h (Figure 5a), 48 h (Figure 5b), and 72 h (Figure 5c) compared to the control for all doses analyzed (*p* > 0.05). However, VERO was sensitive to the Bc treatment at 24 and 48 h (Figure 5d,e). It is noteworthy that the decrease below 70% in the mean fluorescence only occurs at the highest dose at 48 h (Figure 5e) with a viability recovery profile at 72 h (Figure 5f).

**Figure 5:**
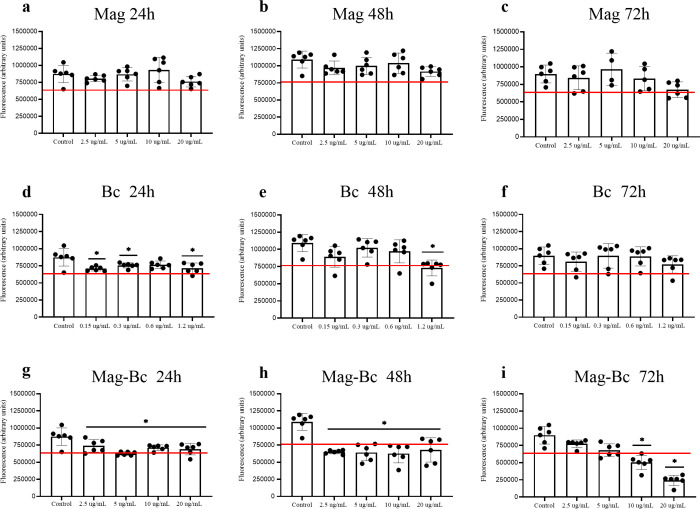
VERO cells exposed to the Mag, Bc, and Mag-Bc005 samples. Cell viability was evaluated by CellTiter-Blue in VERO cells exposed to Mag (a–c), Bc (d–f), and Mag-Bc005 (g–i) for 24, 48, and 72 h at the subsequent concentrations 2.5, 5, 10, and 20 μg.mL^–1^ and the adjusted doses to 6% of the equivalent one for the Bc group. Results represent the fluorescence mean ± SD of the sextuplicate. *Statistical difference in relation to the control group (*p* < 0.05). A red line was used to identify the 70% viability limit according to ISO10993-5.

VERO was the cell line most sensitive to the nanocomplex (Figure 5g–i). After 24 and 48 h of exposure (Figure 5g,h), a reduction in the fluorescence was observed for all doses evaluated (*p* < 0.05). However, the mean fluorescence recorded for all doses at 24 h of exposure was equivalent to at least 70% of the control fluorescence, unlike at 48 h. At 72 h (Figure 5i), VERO showed a viability recovery profile for the two lowest doses while maintaining the dose response for the two highest doses with fluorescence below 70% of the control's one (*p* < 0.05).

The AML-12 cell line, a murine hepatocyte model, when exposed to the uncoated nanoparticle, Mag, showed no reduction in viability at 24 h (Figure 6a), 48 h (Figure 6b), and 72 h (Figure 6c) compared to the control for all doses analyzed (*p* > 0.05). This profile was also recorded for these cells when exposed to the BC treatment, which showed no reduction in mean fluorescence below 70% of the control fluorescence at all exposure times, despite a decrease in mean fluorescence (*p* < 0.05). For the nanocomplex treatment, the AML-12 results were closer to the Bc treatment. A resistant profile was noted in the beginning, with fluorescence decreasing at 72 h for all doses analyzed (*p* < 0.05). It is worth noting that only the highest dose at 48 h shows fluorescence reduction below 70% compared to the control group.

**Figure 6:**
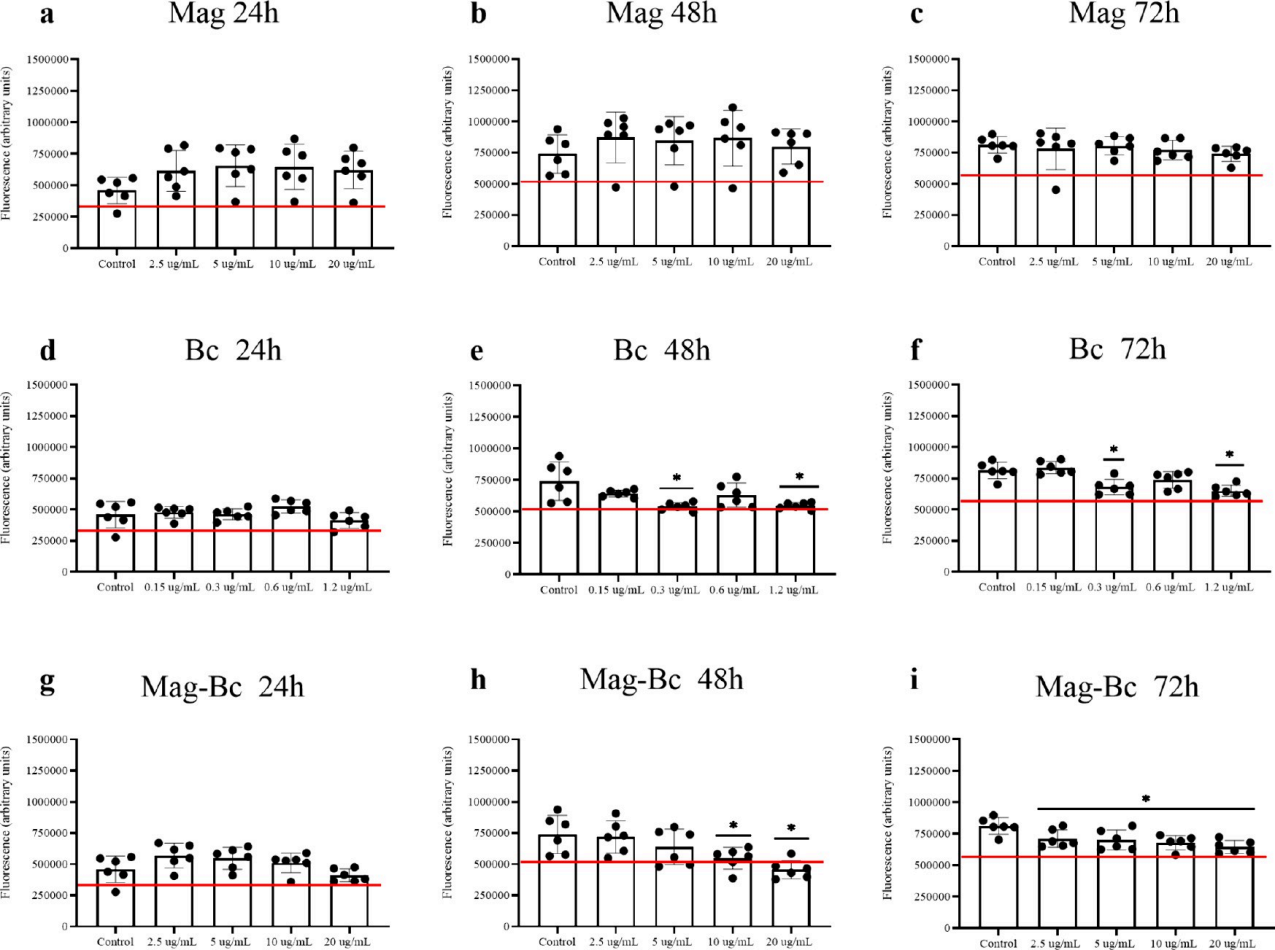
AML-12 cells exposed to Mag, Bc, and Mag-Bc005 samples. Cell viability was evaluated by CellTiter-Blue in AML-12 cells exposed to Mag (a–c), Bc (d–f), and Mag-Bc005 (g–i) for 24, 48, and 72 h at the subsequent concentrations 2.5, 5, 10, and 20 μg.mL^–1^ and the adjusted doses to 6% of the equivalent one for the Bc group. Results represent the fluorescence mean ± SD of the sextuplicate. *Statistical difference in relation to the control group (*p* < 0.05). A red line was used to identify the 70% viability limit according to ISO10993-5.

In this study, Mag and Bc groups were used as controls, and the results obtained were compared with the ISO10993-5 standard, which states that a substance is considered cytotoxic when cell viability is less than 70% compared to a control group.^40^ VERO and AML-12 cell lines were chosen for this study to evaluate possible systemic toxicity, highlighting their original roles in the kidneys and liver. In this study, these cells demonstrated a dose-dependent response, decreasing fluorescence compared to that of the control group. However, only the high doses tested for the nanocomplex showed a reduction in viability under 70% for the VERO cell line in two exposure times.

These results align with other studies using these cell lines, which have shown a dose–response decrease in viability when exposed to different types of nanoparticles.^41–43^ IONPs are known for biocompatibility, as demonstrated by these cells in this study. However, iron accumulation could increase ROS formation, triggering oxidation processes, leading to cell death.^4,44^ Establishing the cytotoxic profile of a nanomaterial, using different types of cell lines, could help to understand the potential toxicity *in vivo* since viability results can vary based on the concentration, exposure time, and type of cells.^41,43,45^

TM3, a murine Leydig cell, when exposed to the uncoated nanoparticles, showed no reduction in viability at 24 h (Figure 7a) and 48 h (Figure 7b), compared to the control (*p* > 0.05). A dose-dependent reduction in the mean fluorescence was observed for TM3 compared to the control group at 72 h (Figure 7c) (*p* < 0.05). However, the mean fluorescence recorded for all doses was equivalent to at least 70% of the fluorescence recorded for the control. For the treatment with Bc shown in Figure 7d–f and with the nanocomplex in Figure 7g–i, despite the decrease in the mean fluorescence recorded for the two highest doses, TM3 did not show any mean fluorescence values below 70% of the control group at 24, 48, and 72 h.

**Figure 7:**
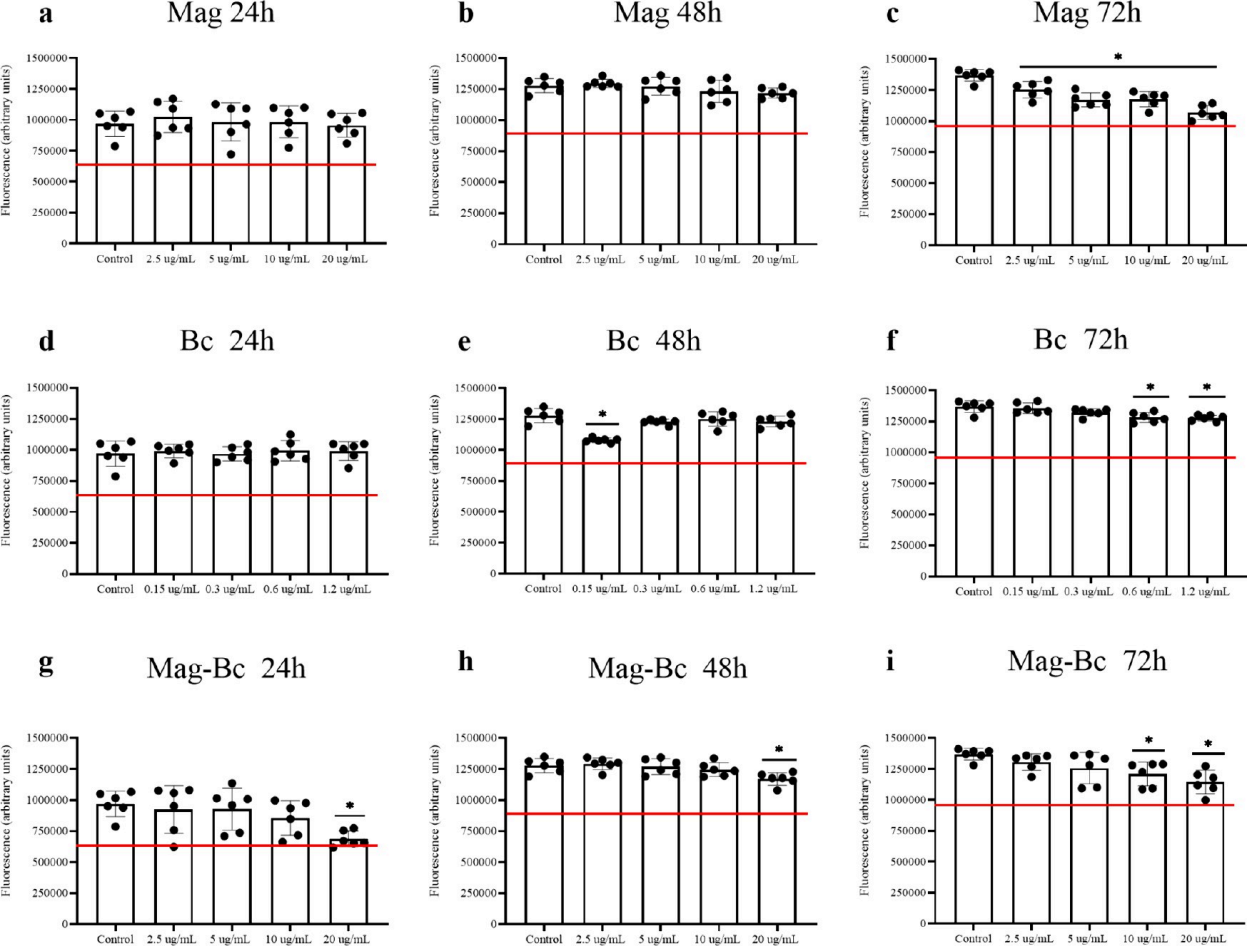
TM3 cells exposed to Mag, Bc, and Mag-Bc005 samples. Cell viability was evaluated by CellTiter-Blue in TM3 cells exposed to Mag (a–c), Bc (d–f), and Mag-Bc005 (g–i) for 24, 48, and 72 h at the subsequent concentrations 2.5, 5, 10, and 20 μg.mL^–1^ and the adjusted doses to 6% of the equivalent one for the Bc group. Results represent the fluorescence mean ± SD of the sextuplicate. *Statistical difference in relation to the control group (*p* < 0.05). A red line was used to identify the 70% viability limit according to ISO10993-5.

The TM3 cell line was selected as the target cell line from male reproductive tissue due to the role of Leydig cells in the maintenance of spermatogenesis^11,46^ and the current studies with Leydig cell physiology and IONPs.^9,10^ Leydig cells appear to be more sensitive to IONPs than other testicular cells, as seen *in vitro* ^10^ and *in vivo*.^9^ In these studies, the authors highlight the high uptake of these nanoparticles by Leydig cells, leading to some physiological alterations. Previous studies have shown that the cellular response, such as changes in viability or metabolic activity, generally increases with nanoparticle dose.^47,48^

In the dose range evaluated in this study, TM3 cells were more tolerant when exposed to the nanocomplex than were the kidney and liver cells. This Leydig cell line shows some plasticity in the cell response when they are exposed to different types of nanoparticles. The cytotoxic profile of different inorganic nanoparticles has been evaluated using the TM3 cell line, being exposed to gold (AuNp)^48^ and titanium (TiNp)^47^ nanoparticles as an example. In general, a dose-dependent response was observed for these inorganic nanoparticles with treatment doses varying from 12.5 to 100 μg.mL^–1^. Physicochemical properties, such as chemical composition, particle morphology, and size, can influence biocompatibility, as seen among these studies.

Nanoparticle coating/functionalization has several applications, like improving their stability, increasing biocompatibility, and targeting, where coatings can carry bioactive molecules or ligands.^8,49^ IONPs are generally coated to reduce aggregation and cytotoxicity. For all cell lines investigated in this study, it was possible to see some similarities between the cell response when treated with the nanocomplex and Bc, reinforcing the functionalization and stability of Bc on magnetite. Therefore, to evaluate the nanoparticle uptake, superoxide detection, testosterone secretion, and cellular growth on the TM3 cell line, a target dose of 10 μg.mL^–1^ over 48 h of exposure was chosen. This target dose was the highest one that showed no effect on cell viability at two exposure times (24 and 48 h), choosing the longest one for subsequent experiments.

### 2.6 Nanoparticle Uptake and Intracellular Iron Detection

Nanoparticles were observed within the cytoplasm of TM3 cells following treatment with Mag (Figure 8a–c) and Mag-Bc (Figure 8d–f). Notably, the uptake of both coated and uncoated nanoparticles was evident, suggesting that the Bc coating did not hinder cellular internalization, a finding supported by Transmission Electron Microscopy (TEM) images. The nanoparticles manifested as electron-dense structures localized within endosomes (Figure 8, yellow arrowheads) with no such structures identified in the control cells (Figure 8g–i). As previously reported, endocytosis represents one of the primary internalization pathways for iron oxide nanoparticles (IONP).^50^ Additionally, the existing literature indicates that TM3 cells possess the capability to internalize various types of nanoparticles, for which TEM is often employed as a characterization tool.^45,47,48^ Leydig cells, in particular, exhibit a notable affinity for this class of nanoparticles,^9^ although the underlying mechanisms remain inadequately understood.

**Figure 8:**
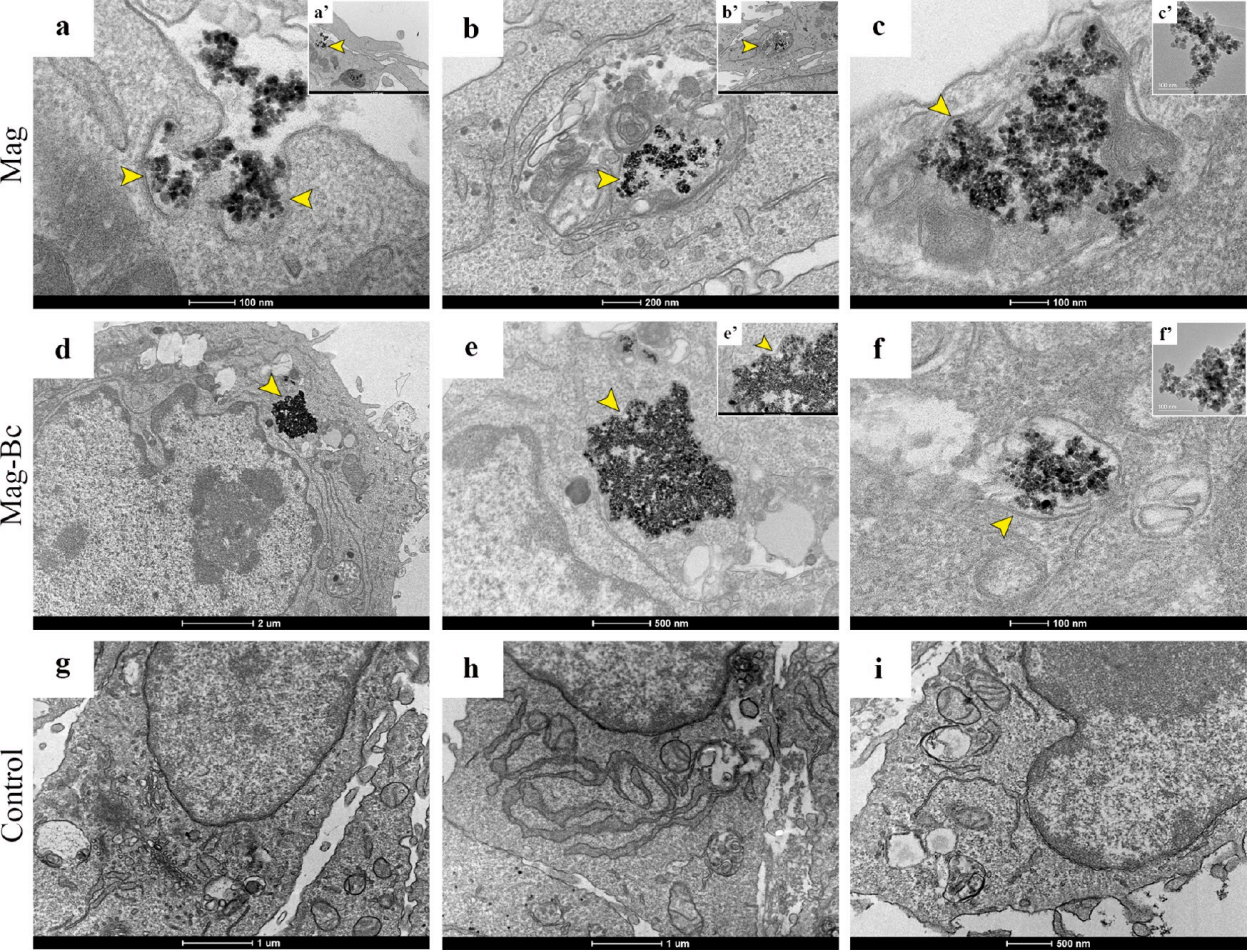
Transmission electron microscopy (TEM) analysis of nanoparticle internalization in TM3 Leydig cells. TM3 cells were exposed to Mag (a–c) or Mag-Bc005 (d–f) at 10 μg·mL^–1^ for 48 h, with untreated cells as control (g–i). (a–c) Magnetite (Mag) internalization: endosomal compartments containing internalized magnetite nanoparticles and cytoplasmic accumulation at varying magnifications (yellow arrowheads indicate endosomal membranes and internalized nanoparticles). (d–f) Mag-Bc005 internalization: endosomal localization of Bc-coated nanoparticles at varying magnifications (yellow arrowheads indicate endosomal membranes and internalized nanoparticles), demonstrating efficient cellular uptake. (g–i) Control: untreated TM3 cells showing absence of electron-dense nanoparticle structures. Scale bars and magnifications are indicated in each image.

Complementing the TEM analysis, Prussian blue staining was performed on TM3 cells exposed to the nanoparticles. The presence of blue staining indicates positive intracellular iron detection. Ferricyanide, which reacts with Fe^3+^ from Mag (Figure 9a–a’’) and Mag-Bc (Figure 9b–b’’), resulted in a bluish stain in the cytoplasm of the cells, 48 h after exposure to a 10 μg.mL^–1^ dose. The control group, in contrast, was only counterstained with neutral red (Figure 9c–c’’), confirming the specificity of the Prussian blue reaction. Our findings align with previous studies reporting iron oxide nanoparticle detection *in vitro,* using this method in different cell types, such as macrophages,^47^ osteoblasts,^48^ and fibroblasts.^42^ Prussian blue staining has been previously used to identify citrate-coated iron oxide nanoparticle uptake by Leydig cells both *in vitro* ^10^ and *in vivo*.^9^ In this study, this technique confirmed iron detection in TM3 cells exposed to Bc-coated magnetite nanoparticles, demonstrating that surface functionalization with benzalkonium chloride did not impair cellular iron detection or nanoparticle internalization.

**Figure 9:**
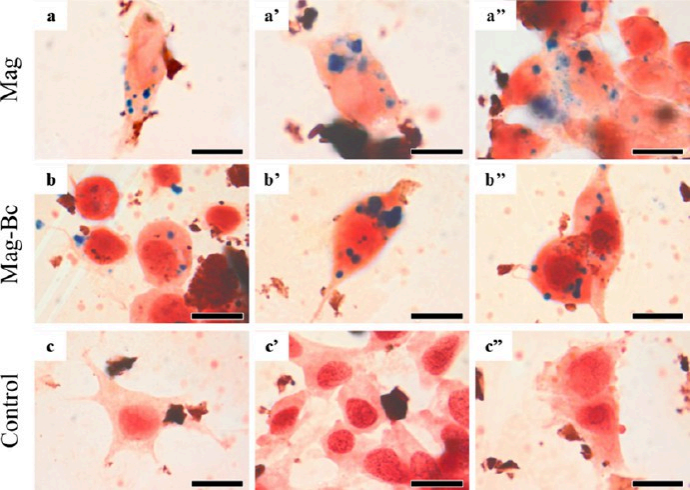
Prussian blue staining of TM3 cells exposed to Mag and Mag-Bc005. Intracellular iron detection by Prussian blue staining (blue) with neutral red counterstaining (red) in TM3 cells after 48 h of exposure to Mag (a–a’’) and Mag-Bc005 (b–b’’) at 10 μg.mL^–1^ and the control group (c–c’’). Scale bar: 10 μm.

### 2.7 Superoxide Detection and Testosterone Secretion

For the TM3, the superoxide anion detection (Figure 10a,b) showed that the nanocomplex has an intermediate response compared to the Mag and Bc groups, leveling the detection of superoxide to basal levels (control group) (*p* > 0.05). Only the uncoated nanoparticle, Mag, showed an increase in superoxide detection (*p* < 0.05), in contrast to the Bc group (*p* < 0.05). MitoSOX is a fluorogenic dye used to detect superoxide anion (O2^•–^) in the mitochondria of living cells.^10^ IONPs are known to increase reactive oxygen species (ROS) in different types of cells,^10,51^ as seen in this study for the uncoated nanoparticle. The increase in ROS was also reported for TM3 cells after exposure to different types of nanoparticles.^47,48,52^ Benzalkonium chloride is known in high concentrations to lead the cells to increase ROS production.^53^ However, it has been demonstrated that Bc can have an antagonistic dose–response to ROS production.^54^

**Figure 10:**
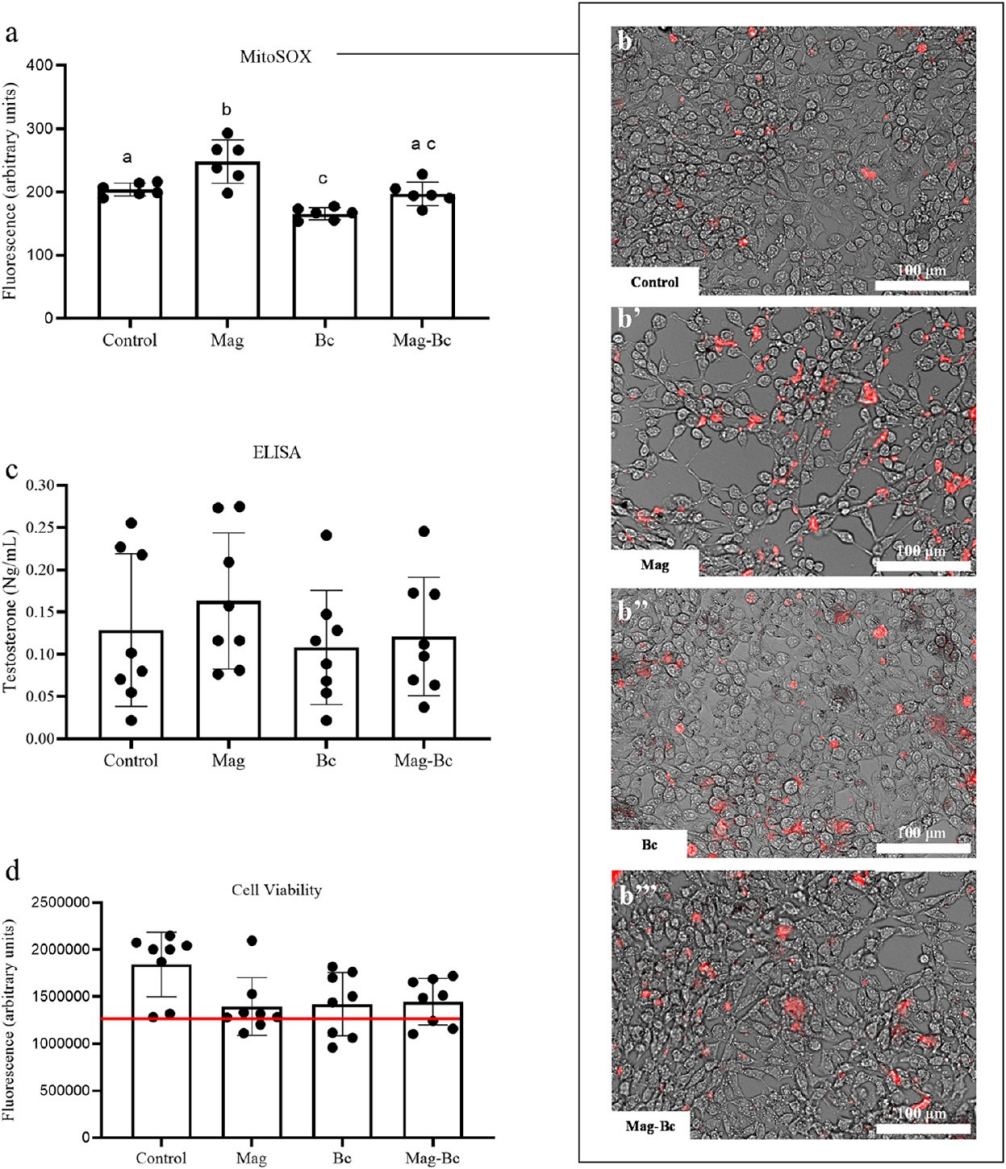
Mitochondrial superoxide production and testosterone levels in TM3 cells exposed to Mag, Bc, and Mag-Bc005. (a, b) MitoSOX assay in TM3 cells exposed to Mag and Mag-Bc005 at 10 μg.mL^–1^ and Bc at 0.6 μg.mL^–1^ at 48 h. (a) Quantitative data of mitochondrial superoxide anion (O2^•–^) production. Statistical differences expressed by different letters (*p* < 0.05). (b–b’’’) Fluorescent images of TM3 cells exposed to MitoSOX Red superoxide indicator. (c) ELISA data for the TM3 cells exposed to Mag and Mag-Bc005 at 10 μg.mL^–1^ and Bc at 0.6 μg.mL^–1^ after 48 h for testosterone evaluation. (d) Cell viability was evaluated by CellTiter-Blue in the TM3 cell culture used for the ELISA assay. *Statistical difference in relation to the control group (*p* < 0.05). A red line was used to identify the 70% viability limit according to ISO10993-5.

Superoxide production was investigated in this study due to the possibility of interaction between the Mag and Mag-Bc005 with mitochondria. Mitochondrial toxicity can inhibit several cellular processes,^51^ including testosterone production in Leydig cells.^55^ The TM3 testosterone secretion (Figure 10c) and the cell viability on the cells tested on the hormonal assay (Figure 10d) showed no statistical differences (*p* > 0.05) among all the groups compared to the control (*p* > 0.05). This contrasts with other results showing a reduction in testosterone levels for Leydig cells *in vivo* ^9^ and a reduction in 3β-HSD, the enzyme that catalyzes steroid biosynthesis^10^ after exposure to IONPs *in vitro*. Although it does not increase superoxide anions in mitochondria, additional studies (*in vivo*) are needed to verify whether the nanocomplex can interfere with androgen synthesis. Nanocomplex, in this context, appears to be more biocompatible at the target dose under study, showing a good response by TM3 cells at 48 h.

### 2.8 Cellular Growth

After the cytotoxic profile of the nanocomplex was established, the TM3 cell growth was evaluated, as shown in Figure 11. Overall, the cells exposed to the nanocomplex (green line) and Bc (blue line) showed a slow growth from day 3 until day 8 compared to the control group (gray line) (*p* < 0.05). The cells treated with Mag (red line) did not show any statistical difference from the control group for all experimental days (*p* > 0.05). Regarding the monolayer appearance (Figure 12a–d), on day 6, the control (Figure 12a’’) and Mag (Figure 12b’’) groups reached high confluence with few empty spaces containing isolated cells, unlike the monolayer appearance of the cells exposed to the Bc (Figure 12c’’) and nanocomplex (Figure 12d’’), which did not reach high confluence and contained more isolated cell spots until day 8 (Figure 12c’’’,d’’’).

**Figure 11:**
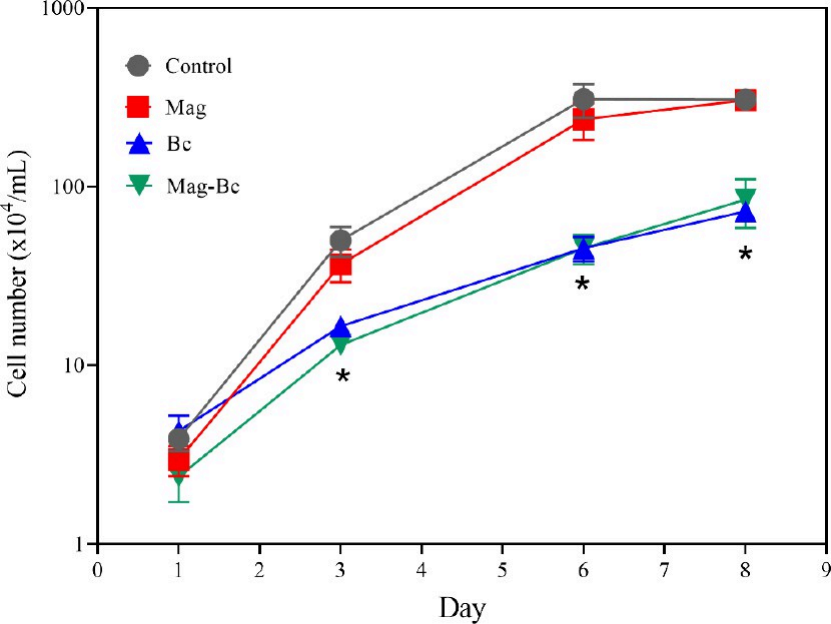
TM3 cell proliferation assessment following exposure to the Mag, Bc, and Mag-Bc005 samples. Growth curve for the TM3 cell line after exposure to Mag (red), Mag-Bc005 (green) at 10 μg.mL^–1^, and Bc (blue) at 0.6 μg.mL^–1^ compared to the control group (gray). *Statistical difference in relation to the control group (*p* < 0.05).

**Figure 12:**
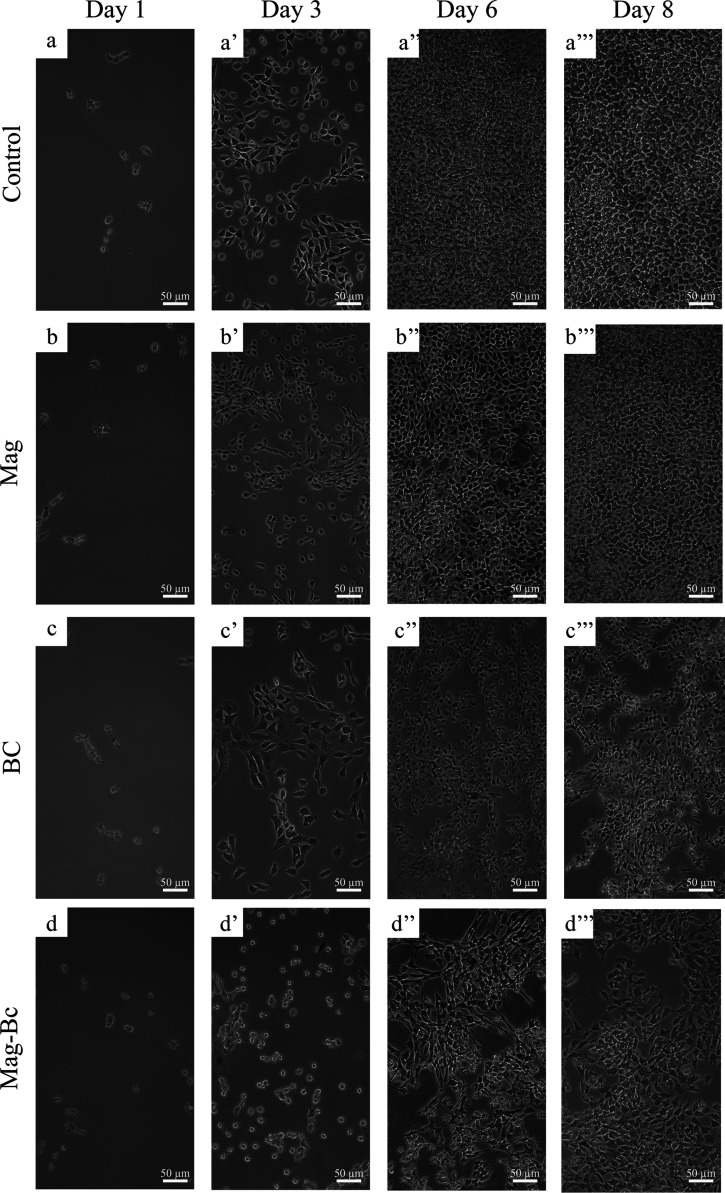
Cellular confluence evolution in TM3 cells exposed to Mag, Bc, and Mag-Bc005. Monolayer appearance for TM3 cells of the control (a–a’’’), Mag (b–b’’’), Bc (c–c’’’), and Mag-Bc005 (d–d’’’) groups from day 1 to day 8. Reduced cellular confluence is observed on days 6 and 8 for the Bc and Mag-Bc005 groups.

Cell growth is an important parameter in the study of cellular health in response to nanomaterial exposure.^47,52,56,57^ These results suggest an arrest in TM3 growth, probably by the presence of benzalkonium chloride, since the Bc and Mag-Bc005 groups showed the same pattern of growth. Bc could be negatively affecting the cell cycle after 72 h of exposure. In contrast, the Mag group showed growth similar to that of the control group. Negative effects of IONP functionalization in the testicular environment were also reported by Dantas et al.^10^ for citrate-coated IONPs. The arrest in Leydig cell growth caused by the new nanocomplex has the potential to be applied in developing gonads to reduce the number of Leydig cells and consequently hinder gamete production.

## 3 Conclusions

Physicochemical characterization confirmed the successful creation of partially oxidized magnetite with an effective Bc coating. Zeta potential, saturation magnetization, and FTIR data show the stability of the nanocomplex. Viability assays indicated that VERO and AML-12 cells were more sensitive to Mag-Bc than TM3 Leydig cells, suggesting better biocompatibility with testicular cells. At the target dose, Mag-Bc maintained TM3 cell viability and testosterone synthesis without inducing mitochondrial stress over 48 h, indicating no disruption of endocrine function. However, prolonged exposure led to growth arrest in TM3 cells, implying that chronic exposure may affect Leydig cell proliferation. Overall, the Mag-Bc nanocomplex shows a favorable safety profile for testicular applications, warranting further *in vivo* studies to assess its potential for controlled Leydig cell suppression.

## 4 Experimental Procedure

### 4.1 Reagents

For the synthesis of magnetic nanoparticles, the following high-purity grade reagents were employed: iron­(III) chloride hexahydrate (Sigma-Aldrich, Riedel-de Haen, So̷borg, Denmark), sodium sulfite (Sigma-Aldrich, Riedel-de Haen, So̷borg, Denmark), ammonium hydroxide (Fluka, Seelze, Germany), aqueous tetramethylammonium pentahydrate hydroxide solution at 25% (TMAOH, Sigma-Aldrich, So̷borg, Germany), and hydrochloric acid (Sigma-Aldrich, Riedel-de Haen, So̷borg, Denmark). Benzalkonium chloride (Bc) was used for coating (Exodo Científica, São Paulo, Brazil).

For cell culture assays, the following reagents were used: minimum essential medium (α-MEM, Nova Biotecnologia, Brazil), Dulbecco’s modified Eagle medium:nutrient mixture F12 (DMEM:F12, Gibco, Thermo Fisher Scientific, USA), fetal bovine serum (FBS, Nova Biotecnologia, Brazil), equine serum (ES, Nova Biotecnologia, Brazil), insulin–transferrin–selenium (Sigma-Aldrich, Gaithersburg, MD, USA), penicillin–streptomycin (Sigma-Aldrich, Gaithersburg, MD, USA), phosphate buffered saline (PBS, Nova Biotecnologia, Brazil), trypsin-EDTA 0.05%, (Gibco, Thermo Fisher Scientific, USA), CellTiter-Blue (Promega, Madison, USA), Triton X-100 (Sigma-Aldrich, Gaithersburg, MD, USA), neutral red—Neutralrot (Merck, Darmstadt, Germany), potassium ferrocyanide (VETEC, São Paulo, Brazil), and Luria–Bertani broth medium (HiMedia, India).

### 4.2 Synthesis of Magnetite and Bc-Coated Magnetite Nanoparticles

The method of coprecipitation described^30,58,59^ was adopted here. In brief, sodium sulfite (10 mmol) and ferric chloride hexahydrate (30 mmol) were dissolved in a HCl solution (0.5 mol L^–1^) and mixed until the color of the resulting solution changed from dark yellow to light yellow. A diluted ammonia solution was then added to form a black precipitate. The suspension was stirred for an additional 20 min. After this reaction time, the solution was decanted, allowing the particles to be washed with degassed deionized water. This procedure was repeated three times, and the particles were separated by a permanent magnet afterward. The obtained precipitate was labeled as “Mag”. Benzalkonium chloride (Bc), 100 mg, was combined with varying amounts of Mag to create different proportions: Mag-Bc 1:0.05 (2 g of Mag to 100 mg of Bc) and Mag-Bc 1:0.1 (1 g of Mag to 100 mg of Bc). These samples were labeled as “Mag-Bc005” and “Mag-Bc01”, respectively.

### 4.3 Nanoparticle Characterization

Different techniques were employed to identify and characterize the material properties. The morphology, homogeneity, and size of the samples were observed using SEM (FEG SIGMA VP, Zeiss Microscopy) and TEM (Tecnai G2-20, FEI SuperTwin 200 kV). Before SEM analysis, the samples were fastened to a sample holder with the help of a double carbon ribbon and covered with gold. SEM images were obtained, and additionally elemental analysis was performed by EDS. For the TEM analysis, 10 μL of a 1 mg/mL suspension of the nanoparticles was placed on a carbon-coated Cu-TEM grid. The TEM images were used to measure particle size and to establish the size distribution, the HRTEM images were utilized to check the crystallographic planes, and the SAED patterns were collected to identify the crystalline phase.

The powder XRD analysis was carried out on a diffractometer (Rigaku, DMAX-Ultimate-Cu Kα radiation, 40.0 kV, 20.0 mA, and continuous scanning range from 15.0 to 70.0° 2θ). Phase identification was performed using Jade + software (Materials Data, Inc.). The Rietveld structural refinement was performed by using the FULLPROF 2013 program. Mössbauer spectra of ^57^Fe were recorded in transmission geometry employing a ^57^Co/Rh source calibrated with reference to α-Fe. Measurements were conducted both at room temperature and at 77 K in the absence of an externally applied magnetic field.

FTIR spectra were obtained in a Bruker Vertex 70v spectrometer (Billerica, MA, USA) equipped with an ATR-Platinum accessory with a diamond crystal. For these analyses, samples were pressed with KBr (approximately 1%) and analyzed in the transmission mode. X-ray fluorescence was used with a high-frequency (4 kW) dispersive X-ray fluorescence spectrometer (Rigaku, Model NEX CG). The magnetic properties of the samples were characterized using a vibrating sample magnetometer (Lake Shore 7400 Series) instrument at room temperature. Magnetic hysteresis loops were measured up to a maximum applied field of 1.8 T (18 kOe).

The XPS was employed to characterize the surface composition and chemical environment of the Mag-Bc005 nanocomplex. The analysis was performed to determine the chemical states and to estimate the surface atomic concentrations of the elements present on the samples. The spectra were acquired using a UHV Specs surface analysis system equipped with a Phoibos-150 electron analyzer and a monochromated Al Kα radiation source (1486.6 eV, 300 W). An electron flood gun operating at 0.1 μA was employed to minimize surface charging effects during the measurement. The C 1s peak at 284.6 eV was used as an internal reference for calibration of the binding energies of the detected elements. The experimental spectra were fitted by using CasaXPS software.

The ZP and DLS measurements were carried out using a Litesizer 500 particle analyzer (Anton Paar GmbH, Austria) equipped with a 658 nm wavelength laser. Analyses were performed at 25 ± 2 °C, using diluted colloidal suspensions in filtered deionized water. Thermogravimetric analyses, including TGA and DTG, were also conducted to determine the composition of the Bc coating on the coated samples. TGA thermograms were obtained with a DTG60H Shimadzu analyzer under an air flow rate of 20 mL min^–1^ with a heating rate of 20 °C min^–1^ up to 600 °C, an alumina crucible, and with a sample mass of approximately 10.0 mg.

### 4.4 Biological Assays and Sample Preparation

For biological assays, the samples were sterilized and prepared following standardized procedures. The main solution of Bc was diluted in sterile water for injection and then further diluted to the desired concentrations. For the biological assays, Mag-Bc005 was the nanocomplex used since the sample exhibited the best magnetization values (see Results and Discussion section). The nanoparticles (Mag and Mag-Bc005) were subjected to ultraviolet (UV) radiation for 30 min for sterilization to ensure microbiological safety. Afterward, Mag and Mag-Bc005 were dispersed in sterile water for injection and homogenized in a sonicator for 24 min to obtain the main suspension used in the *in vitro* studies, according to predefined concentrations.^41,42,48^

To evaluate UV radiation efficiency, the nanoparticle suspension was tested for bacterial growth.^60^ For that, 100 μL of nanoparticle suspension at 10 μg.mL^–1^ irradiated by UV for 30 min was inoculated into sterile tubes containing 10 mL of LB medium. Tubes only with LB medium were used as a control, and 100 μL of *Escherichia coli* (44-0300, One Shot TOP10F’ Chemically Competent Cells, Thermo Fisher Scientific, USA) bacteria was used as a positive control (ISO 21149:2017). The LB medium was previously prepared and autoclaved. The samples were incubated in a Shaker (Major Science Co., Ltd., Taiwan) at 180 rpm and 37 °C in triplicate. After incubation, the medium was evaluated for turbidity and precipitation, and the readings were performed using the plate reader Synergy 2 (BioTek, USA, 600 nm) at 24 and 48 h.^61^

### 4.5 Cell Culture

To evaluate the cytotoxic profile of the nanocomplex on the TM3 (CRL-1714) cell line, a murine Leydig cell model, the results of viability were compared to the AML-12 (CRL-2254) cell line, a murine hepatocyte model, and the VERO (CCL-81) cell line, derived from kidney tissue of a normal, adult African green monkey. These cell lines were chosen because they have been used in nanotoxicology studies and to infer comparatively possible systemic and testicular effects of the nanocomplex exposure.^41–43,47,48^

#### 4.5.1 TM3 Cell Culture

The TM3 cell line was obtained from ATCC (Arlington, VA, USA). After thawing, cells were cultured in a DMEM:F12 medium supplemented with 5% horse serum, 2.5% fetal bovine serum, and 1% penicillin–streptomycin. Cultures were maintained at 37 °C in a humidified 5% CO_2_ atmosphere. Subculturing was performed at a 1:80 ratio, with medium renewal every 3 to 4 days. Cells were used up to passage 6.^62^

#### 4.5.2 AML-12 Cell Culture

The AML-12 cell line was obtained from ATCC (Arlington, VA, USA). After they were thawed, cells were cultured in DMEM:F12 supplemented with 10% fetal bovine serum, 10 μg.mL^–1^ insulin, 5.5 μg.mL^–1^ transferrin, 5 ng.mL^–1^ selenium, 40 ng.mL^–1^ dexamethasone, and 1% penicillin–streptomycin. Cultures were maintained at 37 °C in a humidified atmosphere of 5% CO_2_. Subculturing was performed at a 1:4 ratio, with medium renewal 2 to 3 times a week. Cells were used up to passage 19.^63,64^

#### 4.5.3 VERO Cell Culture

The VERO cell line was obtained from ATCC (Arlington, VA, USA). After they were thawed, cells were cultured in a MEM medium supplemented with 10% fetal bovine serum and 1% penicillin–streptomycin. Cultures were maintained at 37 °C in a humidified 5% CO_2_ atmosphere. Subculturing was performed at a 1:5 ratio, with medium renewal 2 to 3 times a week. Cells were used up to passage 29.

### 4.6 Cell Viability

To evaluate the cytotoxic profiles of Mag, Bc, and Mag-Bc, the CellTiter-Blue cell viability assay was performed (Promega Corporation, USA). After cell growth and expansion, TM3, AML-12, and VERO cells (1.0 × 10^4^ per well) were seeded in 96-well plates for 24 h. Mag, Bc, and Mag-Bc were tested at concentrations of 2.5, 5, 10, and 20 μg.mL^–1^. For the Bc treatment, the doses used were adjusted to 6% of the previous ones (see the Thermogravimetric Analysis (TGA) section). A viability control, which received only sterile water for injection, and a toxicity control, exposed to 0.1% v/v Triton X-100 for 5 min, were also included.

The assay was conducted at three time points (24, 48, and 72 h). Each well had a final volume of 200 μL. On the assay day, 20 μL of the medium was removed and replaced with 20 μL of the CellTiter-Blue reagent. The plates were incubated for 4 h at 37 °C in a 5% CO_2_ humidified atmosphere, and fluorescence readings were performed using the Cytation 5 Cell Imaging Reader (BioTek, USA, excitation: 560/20 nm, emission: 590/20 nm). The assay was performed in biological sextuplicate, and the mean fluorescence for each sample group was normalized following the CellTiter-Blue protocol. The fluorescence for each group was compared with the control group to assess the cell viability.

### 4.7 Nanoparticle Uptake and Intracellular Iron Detection

#### 4.7.1 Transmission Electron Microscopy

TEM was used to evaluate the nanoparticle uptake. TM3 cells were cultured in a DMEM/F12 medium in a T75 flask for cell culture. After reaching a confluence of 70%, cells were treated with Mag or Mag-Bc samples at a concentration of 10 μg.mL^–1^ for 48 h. After incubation, the monolayer was washed twice with 1x PBS and fixed with 2.5% glutaraldehyde in 0.1 M sodium phosphate buffer, pH 7.4, for 30 min. The monolayer was collected using a cell scraper, centrifuged, and resuspended in 0.1 M sodium phosphate buffer, pH 7.4. Subsequently, secondary fixation was conducted in 2% osmium tetraxide for 30 min at room temperature. The samples were dehydrated in a graded series of ethanol and acetone. After that, they were infiltrated and embedded in resin for the subsequent obtaining of ultrathin sections. Electron micrographs were obtained using a transmission electron microscope (Tecnai G2-12, Bio TWIN 120 kV, Thermo Fischer Scientific/FEI) at the Microscopy Center of the Universidade Federal de Minas Gerais, Brazil.^45^

#### 4.7.2 Prussian Blue Staining

The Prussian Blue staining method was used to evaluate intracellular iron levels.^45,65^ TM3 cells were cultured in a DMEM/F12 medium and seeded onto sterile coverslips placed in 6-well plates at a density of 2 × 10^4^ cells per well. Cells were treated with Mag or Mag-Bc samples at a concentration of 10 μg.mL^–1^ for 48 h. After incubation, the cells were fixed with 4% paraformaldehyde for 30 min and washed twice with 1x PBS. A Prussian Blue solution, prepared by mixing equal volumes of 13% hydrochloric acid and 10% potassium ferrocyanide, was added to each well and incubated for 30 min. The presence of ferric iron (Fe^3^^+^) was visualized as a blue pigment. Cells were then counterstained with neutral red for 5 min to enhance contrast between cellular structures and iron deposits. Microscopic analysis was performed using an Olympus BX60 optical microscope (Olympus BX60; Olympus Corporation) at 1000× magnification.

### 4.8 Mitochondrial Superoxide Indicator

The intracellular superoxide anion (O_2_^•–^) generation was measured using the MitoSOX Red Mitochondrial Superoxide Indicator kit (Thermo Fisher Scientific, USA), according to the manufacturer’s instructions. A total of 2 × 10^4^ TM3 cells (per well) were seeded in 96-well plates and allowed to adhere for 24 h. For that, TM3 cells were treated with the target dose of 10 μg.mL^–1^ and evaluated 48 h later. After the incubation period, the cells were treated with samples of Mag, Bc, or Mag-Bc. The control group received only sterile water for injection. All cultures were maintained at 37 °C in a humidified atmosphere with 5% CO_2_. The assay was performed in biological sextuplicate, and TM3 cells were analyzed using a Cytation 5 Cell Imaging plate reader (BioTek, USA, excitation: 560/20 nm, emission: 590/20 nm).

### 4.9 Hormonal Assay (ELISA)

The testosterone secretion by TM3 cells was evaluated by a commercial testosterone test kit, using the enzyme immunoassay protocol (Monobind Inc., USA) after 48 h of treatment. A total of 4 × 10^4^ TM3 cells (per well) were seeded in 48-well plates and allowed to adhere for 24 h. After the incubation period, cells were treated with Mag or Mag-Bc samples at a concentration of 10 μg.mL^–1^ and with Bc at a concentration of 0.6 μg.mL^–1^. The control group received only sterile water for injection. All cultures were maintained at 37 °C in a humidified atmosphere with 5% CO_2_. The culture medium was collected, and testosterone was extracted. In addition, a cell viability assay was performed on these cells, as described in the viability section, to determine whether the differences observed in the testosterone concentration were due to the action of the samples studied or changes in cell viability.

For testosterone extraction, an aliquot of 60 μL of the culture medium was resuspended in 30 μL of distilled water and mixed with 1 mL of high-performance liquid chromatography (HPLC)-grade diethyl ether. The tubes were centrifuged at 2700*g* for 20 min at 4 °C, and the resulting supernatant was frozen at −80 °C for 30 min. The liquid phase was evaporated overnight, and the resulting pellet was resuspended in 30 μL distilled water, thoroughly mixed, and subjected to centrifugation at 250*g* for 20 min at 4 °C.^66^ The assay was performed in biological octuplicate, and the absorbance was measured at 450 nm.

### 4.10 Growth Curve Assay

The growth of TM3 cells was evaluated by testing the cell growth curve at a dose of 10 μg.mL^–1^ over different time points (1, 3, 6, and 8 days). A total of 1.3 × 10^4^ TM3 cells (per well) were seeded in 6-well plates and allowed to adhere for 24 h. After the incubation period, the cells were treated with 10 μg.mL^–1^ of Mag, Bc, or Mag-Bc. The control group received only sterile water for injection. All cultures were maintained at 37 °C in a humidified atmosphere with 5% CO_2_, and the culture medium was replaced every 2 days. At the specified time points (1, 3, 6, and 8 days), cell counts were performed by using a hemocytometer to assess growth. The experiment was conducted in biological triplicate. Images of the cell cultures were recorded (three images per group) using a camera (HAYEAR microscope camera) coupled to an inverted microscope.^56,57^

### 4.11 Statistical Analysis

Data were subjected to the ROUT method to identify and remove outliers following the GraphPad Prism recommended parameters. The Kolmogorov–Smirnov normality test was used to evaluate whether the parametric distribution was parametric for choosing the test *a posteriori.* The Parameters were subjected to analysis of variance (ANOVA) for parametric data with Dunnett’s multiple comparisons test *a posteriori*. The Kruskal–Wallis test was used for nonparametric data, with Dunn’s multiple comparisons test *a posteriori.* The multiple comparisons test compares the mean of each column with the mean of a control group. All analyses were performed using the GraphPad Prism 8 program (GraphPad Software, Inc. San Diego, USA), and the significance level considered was *p* < 0.05.

## Supplementary Material



## Data Availability

The data supporting this study are available within the manuscript.

## Notes

Microbiological evaluation of Mag and Mag-Bc005 samples for UV radiation efficiency showing the optical density (OD) at 24 and 48 h; hyperfine parameter values from Mössbauer spectroscopy reinforcing the phase identification obtained in this study (PDF)
