# Supplementary material for: Benzalkonium Chloride-Coated Iron Oxide Nanoparticles: Cytotoxic Potential on Murine Leydig Cells (TM3)
Source: ACS Omega. 2025 Dec 10;10(50):62157–75. doi: 10.1021/acsomega.5c09618 (PMC12750389; doi:10.1021/acsomega.5c09618)
Supplement: Supplementary file 1 [file ao5c09618_si_001.pdf]

Benzalkonium Chloride-Coated Iron Oxide Nanoparticles: cytotoxic potential on murine Leydig cells (TM3)

Pedro Igor Macário Viana [1]; Thalita Marcolan Valverde [1]; Daniele Alves Fagundes [3]; Carla Cristina Martins Silva [1]; Graziela de Paula Ferreira Dantas [1]; Larissa Kennedy Moreira Freitas [1]; Danilo Roberto Carvalho Ferreira [4]; Bruna de Paula Dias [4]; Pedro Lana Gastelois [5]; Clascídia Aparecida Furtado [4]; José Domingos Ardisson [3]; Ângela Leão Andrade [2]; Guilherme Mattos Jardim Costa [1]*

[1] Laboratório de Biologia Celular, Departamento de Morfologia, Instituto de Ciências Biológicas (ICB-UFMG), Universidade Federal de Minas Gerais, Belo Horizonte, 31270-901, MG, Brazil.

[2] Departamento de Química, Instituto de Ciências Exatas e Biológicas (ICEB/UFOP), Universidade Federal de Ouro Preto, 35400-000 Ouro Preto, MG, Brazil.

[3] Laboratório de Física Aplicada, Centro de Desenvolvimento da Tecnologia Nuclear (CDTN/CNEN), Belo Horizonte, 31270-901, MG, Brazil.

[4] Laboratório de Química de Nanoestruturas de Carbono, Centro de Desenvolvimento da Tecnologia Nuclear (CDTN/CNEN), Belo Horizonte, 31270-901, MG, Brazil.

[5] Centro de Desenvolvimento da Tecnologia Nuclear (CDTN/CNEN), Belo Horizonte, 31270-901, MG, Brazil.

* Corresponding author: Guilherme Mattos Jardim Costa - gmjc@ufmg.br

Supplementary material

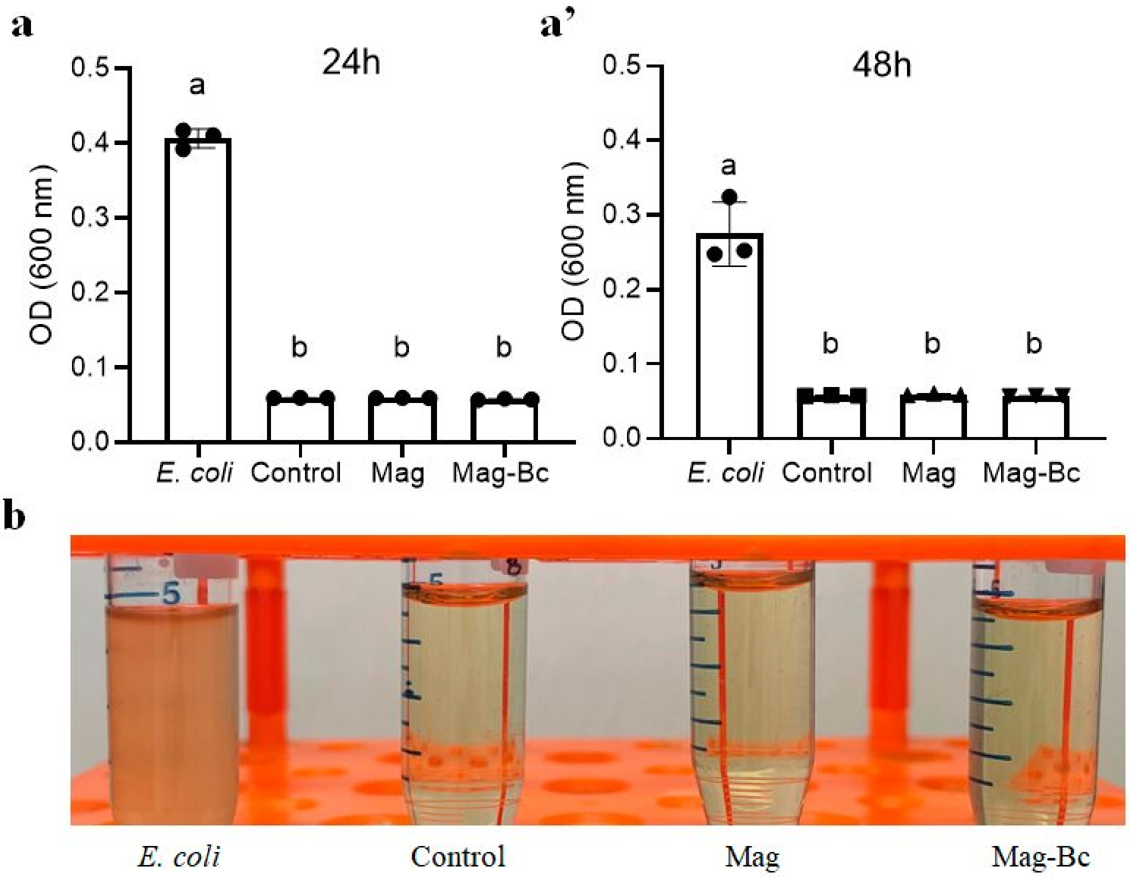

Supplementary Figure 1: Microbiological evaluation of Mag and Mag-Bc005 samples. a-a') optical density (OD) at 24 (a) and 48 (a') hours after incubation. b) Medium turbidity in the positive control *(E. coli),* control, Mag, and Mag-Bc005, after 48 hours of incubation. Statistical differences expressed by different letters ($p<0.05$).

Supplementary Table 1 - Isomer shift (δ), quadrupole displacement (ε), hyperfine magnetic field (BHF), and relative spectral area of Mag nanoparticles at room temperature (RT).

MAG				
**Site**	**δ (±0.05) ($mm.s^{-1}$)**	**ε (±0.05) ($mm.s^{-1}$)**	**B_{HF} (±0.5) (T)**	**Relative spectral area (±1) (%)**
Magnetite (Fe^{3+}; site A)	0.22	0.00	43.0	26
Magnetite (Fe^{2+}/Fe^{3+}; site B)	0.41	0.00	40.5	52
Maghemite	0.33	-0.01	46.1	22

Supplementary Table 2 - Isomer shift (δ), quadrupole displacement (ε), hyperfine magnetic field (BHF), and relative spectral area of Mag-Bc005 nanoparticles at room temperature (RT).

MAG-BC 005				
**Site**	**δ (±0.05) ($mm.s^{-1}$)**	**ε (±0.05) ($mm.s^{-1}$)**	**B_{HF} (±0.5) (T)**	**Relative spectral area (±1) (%)**
Magnetite (Fe^{3+}; site A)	0.30	0.06	40.6	23
Magnetite (Fe^{2+}/Fe^{3+}; site B)	0.31	-0.03	35.9	46
Maghemite	0.29	-0.06	45.2	31

Supplementary Table 3 - Isomer shift (δ), quadrupole displacement (ε), hyperfine magnetic field (BHF), and relative spectral area of Mag-Bc01 nanoparticles at room temperature (RT).

**MAG-BC 01**				
**Site**	**δ (±0.05) ($mm.s^{-1}$)**	**ε (±0.05) ($mm.s^{-1}$)**	**B_{HF} (±0.5) (T)**	**Relative spectral area (±1) (%)**
Magnetite (Fe^{3+}; site A)	0.20	0.00	41.0	19
Magnetite (Fe^{2+}/Fe^{3+}; site B)	0.60	0.00	38.0	38
Maghemite	0.35	0.07	46.3	44

Supplementary Table 4 - Isomer shift (δ), quadrupole displacement (ε), hyperfine magnetic field (BHF), and relative spectral area of Mag nanoparticles at 77 K.

**MAG**				
**Site**	**δ (±0.05) ($mm.s^{-1}$)**	**ε (±0.05) ($mm.s^{-1}$)**	**B_{HF} (±0.5) (T)**	**Relative spectral area (±1) (%)**
Magnetite (Fe^{3+}; site A)	0.34	-0.01	48.6	22
Magnetite (Fe^{3+}; site B)	0.89	0.30	50.2	12
Magnetite (Fe^{3+}; site B)	0.32	-0.02	51.3	33
Magnetite (Fe^{2+}; site B)	0.89	0.50	40.1	7
Magnetite (Fe^{2+}; site B)	1.15	-2.26	39.7	5
Maghemite	0.55	-0.02	51.4	21

Supplementary Table 5 - Isomer shift (δ), quadrupole displacement (ε), hyperfine magnetic field (BHF), and relative spectral area of Mag-Bc005 nanoparticles at 77 K.

MAG-BC 005				
**Site**	**δ (±0.05) (mm.s^{-1})**	**ε (±0.05) (mm.s^{-1})**	**B_{HF} (±0.5) (T)**	**Relative spectral area (±1) (%)**
Magnetite (Fe^{3+}; site A)	0.37	-0.06	47.8	13
Magnetite (Fe^{3+}; site B)	0.75	0.47	49.6	11
Magnetite (Fe^{3+}; site B)	0.27	-0.05	50.7	33
Magnetite (Fe^{2+}; site B)	0.53	0.49	42.7	5
Magnetite (Fe^{2+}; site B)	0.92	-2.20	37.9	2
Maghemite	0.53	-0.01	51.1	36

Supplementary Table 6 - Isomer shift (δ), quadrupole displacement (ε), hyperfine magnetic field (BHF), and relative spectral area of Mag-Bc01 nanoparticles at 77 K.

MAG-BC 01				
**Site**	**δ (±0.05) (mm.s^{-1})**	**ε (±0.05) (mm.s^{-1})**	**B_{HF} (±0.5) (T)**	**Relative spectral area (±1) (%)**
Magnetite (Fe^{3+}; site A)	0.22	-0.07	45.3	11
Magnetite (Fe^{3+}; site B)	0.83	0.50	49.3	11
Magnetite (Fe^{3+}; site B)	0.19	-0.09	50.2	27
Magnetite (Fe^{2+}; site B)	0.78	0.52	42.0	7
Magnetite (Fe^{2+}; site B)	1.20	-2.30	37.4	2
Maghemite	0.50	-0.01	50.5	42
